# Development of Lactic Acid-Fermented Tomato Products

**DOI:** 10.3390/microorganisms8081192

**Published:** 2020-08-05

**Authors:** Annalisa Ricci, Martina Marrella, Jasmine Hadj Saadoun, Valentina Bernini, Francesco Godani, Franco Dameno, Erasmo Neviani, Camilla Lazzi

**Affiliations:** 1 Department of Food and Drug, University of Parma, Parco Area delle Scienze 49/A, 43124 Parma, Italy; annalisa.ricci2@studenti.unipr.it (A.R.); martina.marrella@unipr.it (M.M.); valentina.bernini@unipr.it (V.B.); erasmo.neviani@unipr.it (E.N.); camilla.lazzi@unipr.it (C.L.); 2 Mutti S.pA., Via Traversetolo 28, 43022 Parma, Italy; francesco.godani@muttispa.it (F.G.); franco.dameno@muttispa.it (F.D.)

**Keywords:** lactic acid fermentation, fruit and vegetable extract, tomato juice, tomato puree, DPPH, Folin-Ciocàlteu

## Abstract

Background: lactic acid fermentation was recently proposed to produce fruit and vegetable beverages with high nutritional value. In this study, a wide screening of strains and fermentation parameters was carried out to develop fermented tomato-based drinks containing viable cells and potentially bioactive metabolites. Methods: six different products (three extracts, two tomato juices and one tomato puree) were used as substrate for fermentation. After preliminary testing, eight fermentation conditions for each tested product were selected. The final products were stabilized with pasteurization or refrigeration and further characterized in terms of (i) antioxidant activity and (ii) total polyphenols. Results: selected strains were able to grow in almost all tomato-based products except for one extract. Antioxidant activity and total phenolic content depend on products and fermentation conditions used and, except for tomato puree, an overall increase was observed. The best nutritional profile was reached in fermented samples stored at refrigerated temperature without thermal stabilization. Conclusion: an integrated data vision allowed to choose, for each substrate, the best combination of strains to produce novel fermented tomato-based products with different application perspectives.

## 1. Introduction

Fruits and vegetables are essential sources of water-soluble vitamins (vitamin C and group B), phytosterols, fiber and minerals. Numerous studies have highlighted that their consumption over time help to prevent chronic diseases such as hypertension, vascular and cardiac diseases [1,2,3].

In recent years, lactic acid fermentation of vegetable matrices as a possible strategy for producing beverages that satisfy the needs of the consumers, who increasingly look for ready-to-drink products characterized by high nutritional properties, with health value gaining more attention [4]. Therefore, fruit juices can be the subject of innovation in the production of lacto-fermented drinks characterized by viable microorganisms capable of enriching the intestinal microbiota, and metabolites deriving from fermentation known for their health-promoting effect [5,6]. Lactic acid bacteria (LAB), Generally Recognised As Safe (GRAS), can be responsible for the synthesis of B vitamins, anti-hypertensive peptides, fatty acids, exopolysaccharides [7,8], and the selection of LAB strains to be used as starter for fermentation represents a key points of the finished product’s success.

Fruits and vegetables, due to their low pH, high concentration in sugars and the presence of polyphenols, represent an unfavourable growth environment for starter microorganisms [9,10]. To adapt to these stressful conditions, LAB implement specific metabolic pathways like the utilization of carbon source alternatives to sugars, which affect the profile of compounds obtained in the product, e.g., the content of molecules with antioxidant and antimicrobial activity, or profile of the volatile compounds [11,12].

Tomato is an excellent source of health-promoting compounds due to the balanced content of minerals and antioxidants including vitamins C and E, lycopene, β-carotene, lutein and flavonoids such as quercetin. Studies have shown correlations between tomato consumption and lower risk of certain types of cancer, cardiovascular diseases and age-related macular degeneration [13,14,15,16]. Despite the nutritional properties of tomatoes and the beneficial effects of lactic fermentation found in other juices, there are still limited numbers of a commercial products based on fermented tomato juice on the market.

The present study aimed to find the best processing parameters in order to develop a fermented tomato-based drink with good nutritional characteristics. A screening of different LAB strains, considered as single or co-culture, originating from different sources, was evaluated to assess whether, through lactic acid fermentation, it is possible to obtain a new type of drink by analyzing (i) bacterial growth, (ii) antioxidant activity and (iii) content of polyphenols.

## 2. Materials and Methods

### 2.1. Tomato-Based Substrates

Tomato juices were provided by Mutti, a tomato industrial company, located in Traversetolo (Parma, Italy). Six commercial products belonging to different categories were considered for this study: three fruit and vegetable extracts, two tomato juices and one tomato puree.

The main ingredients of fruit and vegetable extracts are the following: tomato, orange, carrot and ginger for the first extract (CA); yellow tomato, apple, carrot and lemon for the second one (CG); tomato, strawberry, pomegranate and violet carrot (CV) for the third (Table 1). Tomato juices were prepared with the “ciliegino” variety (SPC) and with “pizzutello” (SPP), which differ, in addition to the tomato variety used, in sugar content. For tomato puree (C), the double-concentrated formula was provided. All the samples were stabilized by heat treatment at Mutti company, then stored at room temperature until their use.

### 2.2. Lactic Acid Bacteria Strains

Three bacterial strains isolated from dairy products, belonging to different LAB species, were used for fermentation: *Pediococcus acidilactici* (3992, isolated from Grana Padano cheese), *Lacticaseibacillus casei* (2240, from Parmigiano Reggiano cheese), and *Lacticaseibacillus rhamnosus* (1473, from Parmigiano Reggiano cheese). All the strains belong to the collection of the Department of Food and Drug of the University of Parma. The bacterial stock cultures were maintained as frozen at −80 ^°^C in de Man Rogosa and Sharpe (MRS) broth (Oxoid, Basingstoke, UK) added with 12.5% glycerol (*v*/*v*).

### 2.3. Experimental Plan for the Selection of Fermentation Parameters

**Figure 1:**
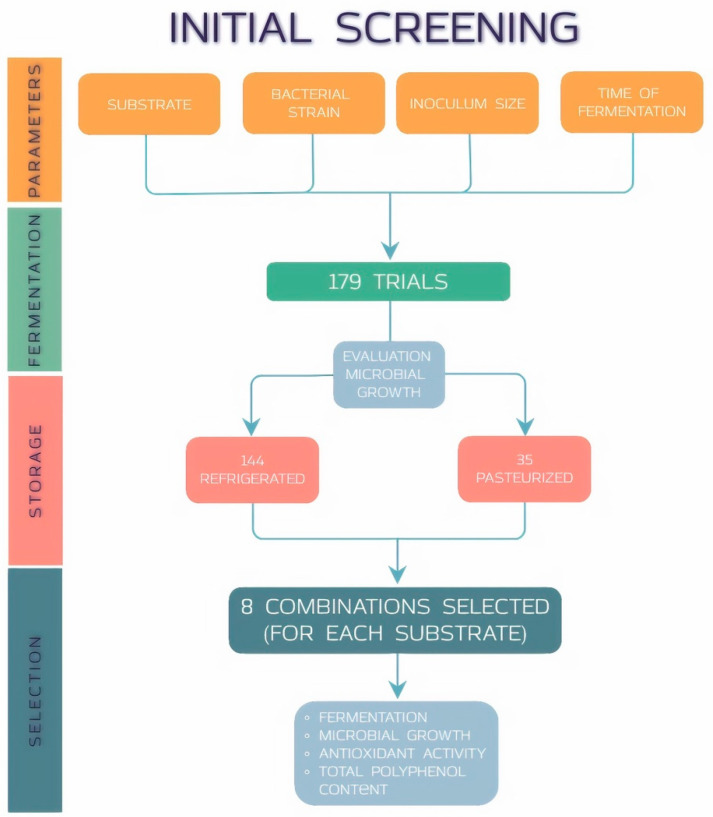
Design of fermentation conditions preliminary screening.

In order to select the optimal fermentation conditions for each of the six substrates considered, a preliminary screening based on 179 trials was carried out (Figure 1). These trials took into account the combination of several factors: (i) type of substrate, (ii) bacterial strains inoculated both as single cultures or in co-culture, (iii) initial inoculum size, (iv) time of fermentation, and (v) type of stabilization after fementation, in particular pasteurization (TT) or refrigeration (NTT). Pasteurization was conducted for 2 min at 100 ^°^C for tomato exctract, while tomato puree and tomato juices were treated for 10 min at 100 ^°^C; refrigeration was set up at 4 ^°^C.

A first sorting was made on the basis of the results of increase of viable cells after fermentation. Bacterial concentration was determined after inoculum (T_0_) and at the established times of fermentation. Ten-fold dilutions in Ringer solution (Oxoid, Basingstoke, UK) were plated in MRS agar (Oxoid), followed by incubation for 48 h in aerobic condition at 37 ^°^C. The pH of all samples was also measured by pH meter (Mettler Toledo, Switzerland) in duplicate at the beginning and at the end of fermentation.

At the end of the selection step, 16 fermentation conditions were chosen for each type of juices, 8 of which fermented and pasteurized, and 8 fermented and refrigerated.

### 2.4. Fermentation Process

Before fermentation, strains were revitalized according to Ricci et al. [17]. The starter inoculum was prepared cultivating the strains overnight (15 h), then cells were harvested by centrifugation (10,000 rpm for 10 min at 4 ^°^C), washed twice with Ringer’s solution (Oxoid, Milan, Italy), and re-suspended in sterile distilled water to reach the final concentration of 9.0 Log CFU/mL. Co-cultures were obtained by mixing single revitalized strains in equal volume and further diluting the mixture to reach the desired concentration in the product. Each culture/co-culture was inoculated into CA, CG, CV, SPC, SPP juices and C puree in different concentrations ranging between 2 Log CFU/mL and 5 Log CFU/mL. Samples were incubated at 37 ^°^C for 6, 14, 24, 30 and 48 h. The initial inoculum and the growth ability after fermentation were checked by plate count agar on (Oxoid) at 37 ^°^C for 48 h.

### 2.5. Measurements of Antioxidant Activity

The antioxidant activity was evaluated by the DPPH radical scavenging activity assay [18]. This spectrophotometric analysis was monitored by measuring the absorbance with a JASCO V-530 spectrophotometer (Jasco Europe, Italy) at a wavelength of 517 nm. Calibration curve was prepared using Trolox as reference standard and results were expressed as mmol/mL Trolox equivalent (TEAC).

### 2.6. Evaluation of Total Phenolic Content

Total phenolic content (TPC) of all samples were analysed by the Folin–Ciocâlteu’s method according to Singleton et al. [19]. The absorbance was monitored by spectrophotometric analysis with a JASCO V-530 spectrophotometer at a wavelength of 760 nm. Calibration curve was prepared using gallic acid (Alfa Aesar, ThermoFischer, Kandel, Germany) as reference compound and results were expressed as mg/L of gallic acid equivalents (GAE).

### 2.7. Statistical Analysis

Analyses were carried out in triplicate. The data are presented as the mean of three replicate ± standard deviation. Independent sample T-test was used to determine significance differences (*p* < 0.05) between fermented samples and the control.

## 3. Results and Discussion

### 3.1. Microbial Growth

**Figure 2:**
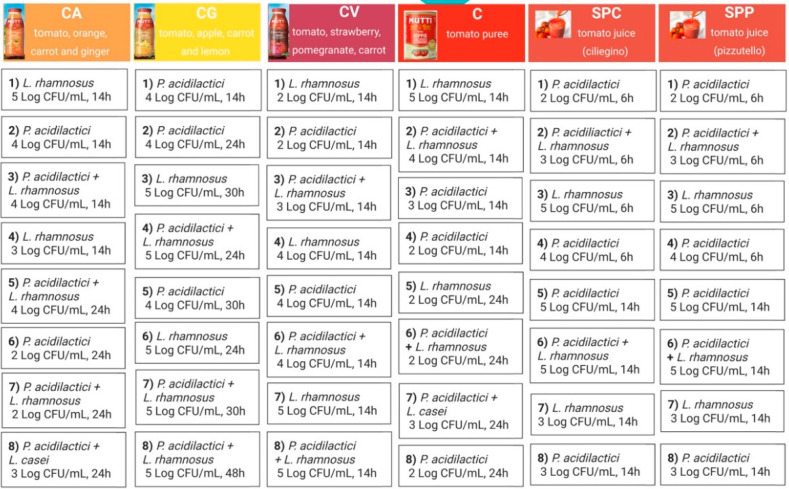
Combination of strains and co-cultures selected for each matrix, inoculum size (Log CFU/mL) and fermentation time. These combinations were used both for pasteurized (TT) and refrigerated (NTT) samples.

In order to identify the optimal fermentation parameters, 179 trials combining single strains and co-cultures, concentration of initial inoculum size, fermentation time and stabilization treatment were performed. Microbial growh ability were evaluated to select the best candidates among all of the tested. A selection of eight conditions for each substrate was carried out based on the results of this parameter (Figure 2). The final fermented products were stabilized with pasteurization or refrigeration and further characterized.

**Figure 3:**
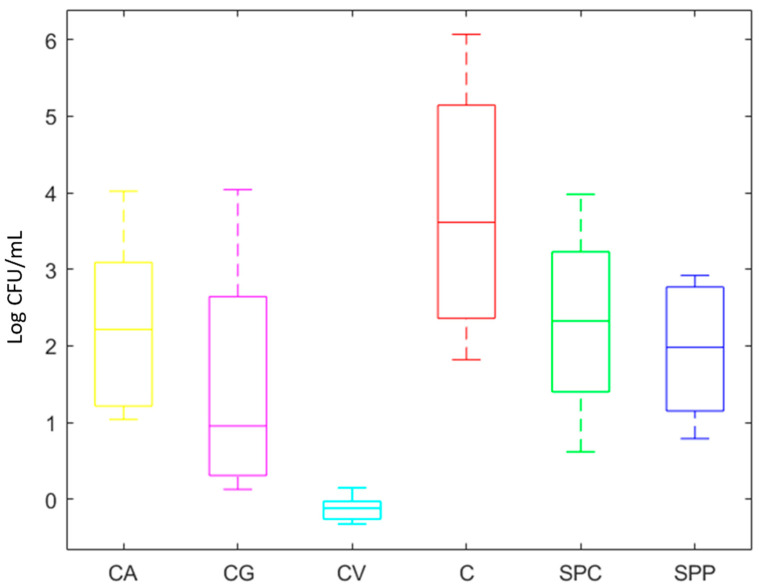
Boxplot representing the increase of viable cell concentration after fermentation in different tomato-based products. The bottom at the end and at the top of whiskers indicates the smallest and the largest results, respectively. The line in the bar, in each plot, is the Median. The x axis reports the products and y axis reports the microbial count expressed as Log CFU/mL.

**Figure 4:**
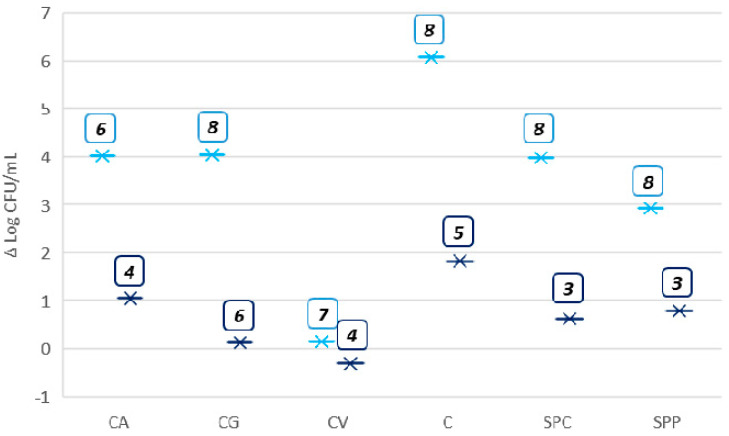
Combinations marked in the boxes showing the highest (light blue) and lowest (dark blue) microbial increase (Δ Log CFU/mL), for each substrates. X axis reports the products and y axis reports the microbial count expressed as Log CFU/mL.

**Figure 5:**
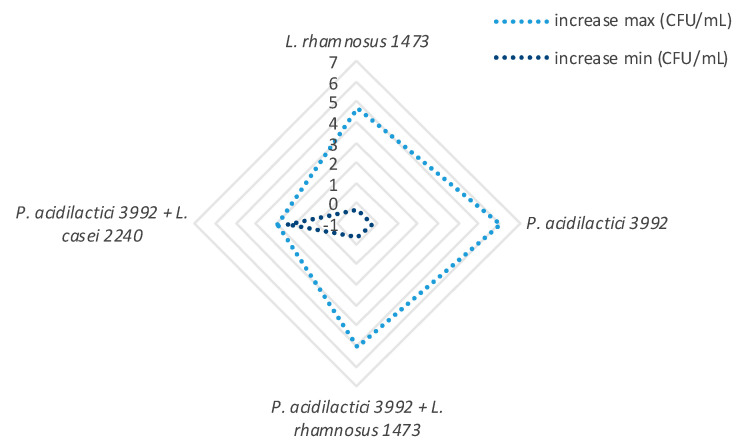
Growth trend of the bacterial strains/co-cultures used. The maximum detected increase is highlighted in light blue and the minimum in dark blue. Bacterial growth is expressed as delta value (Δ-value), as differences from the initial inoculum size and final concentration (from −1 to 7 Log CFU/mL).

The discussion of growth ability was based on the type of substrates used for fermentation (Figure 3), combinations of inoculum size (Log CFU/mL) and fermentation time (Figure 4), and strain species (single and co-cultures) (Figure 5).

Results highlighted that all tested strains grew in tomato-based substrates (Figure 3) except for CV, where any increase of microbial cells number from initial inoculum was observed, probably due to high content of phenolic compounds and low pH values, as reported by Ricci et al. [20] in other fruit juices.

Considering the recently promising performances of strains of dairy origin [11,20,21,22,23], in this study a wide screening of strains and fermentation parameters were carried out in order to develop fermented tomato-based drinks containing viable cells (refrigerated samples) and potentially bioactive metabolites (refrigerated and pasteurized samples).

The highest increase of microbial growth was recorded in matrix C (6 Log CFU/mL), followed by CA, CG and SPC where the increase was closed to 4 Log CFU/mL. Finally, SPP allowed an increase of about 3 Log CFU/mL.

Overall, a heterogeneous growth trend for different combinations was observed even in the same substrate, as highlighted in Figure 4. In particular, the combination no. 6 of CA, with inoculum size of 2 Log CFU/mL of *P. acidilactici* 3992 and 24 h of incubation, reached the highest microbial increase. Otherwise, in the same substrate, the combination no. 4, with inoculum size of 3 Log CFU/mL of *L. rhamnosus* 1473 incubated for 14 h, led to a growth of only 1 Log CFU/mL.

The combination no. 8 of CG, co-culture of *P. acidilactici* 3992 and *L. rhamnosus* 1473, fermented for 48 h, reached the highest bacterial increase (4 Log CFU/mL). The fermentation with monoculture of *L. rhamnosus,* conducted for 24 h with the same inoculum size (combination no. 6), showed a minimum increase (ca. 1 Log CFU/mL).

The combinations no. 8 and no. 3, even with a slightly different trend, turned out to be the best and the worst, respectively, in supporting the microbial growth (Figure 4).

Considering the microbial strains, we observed that *P. acidilactici* 3992 was the strains with highest growth ability, of about 6 Log CFU/mL (Figure 5), but also co-culture of *P. acidilactici* 3992 and *L. rhamnosus* 1473 has proven to be a good starter for tomato products. On the other hand, co-culture of *P. acidilactici* 3992 and *L. casei* 2240 has shown the lowest growth ability.

Despite the microbial growth observed, no significant (*p* > 0.05) reduction in pH were noted in all the samples.

Different authors reported tomato fermentation for different purposes. Some of them apply fermentation as a strategy to recover tomatoes’ by-products for the production of different compounds such as antimicrobials and enzymes using lactic acid bacteria for the fermentation process [17]. Instead, other authors focused their attention on tomatoes juice lacto fermentation using autochthonous strains or reference strains as starters for fermentation [24,25,26,27,28]. In all these studies, lactic acid bacteria had demonstrated to be able to adapt in tomatoes substrate, both juices or paste confirming the results obtained in the present study, in which non-autochthonous strains were adopted. Different tomato varieties were applied as a substrate for fermentation and all supported microbial growth. To the best of our knowledge among the varieties used, those used in this study, ciliegino and pizzutello, were never used as substrate for LAB fermentation. In addition, the mixture of tomato juice with different type of fruit juices was never investigated for the production of a lacto-fermented beverage.

### 3.2. Nutritional Features

**Figure 6:**
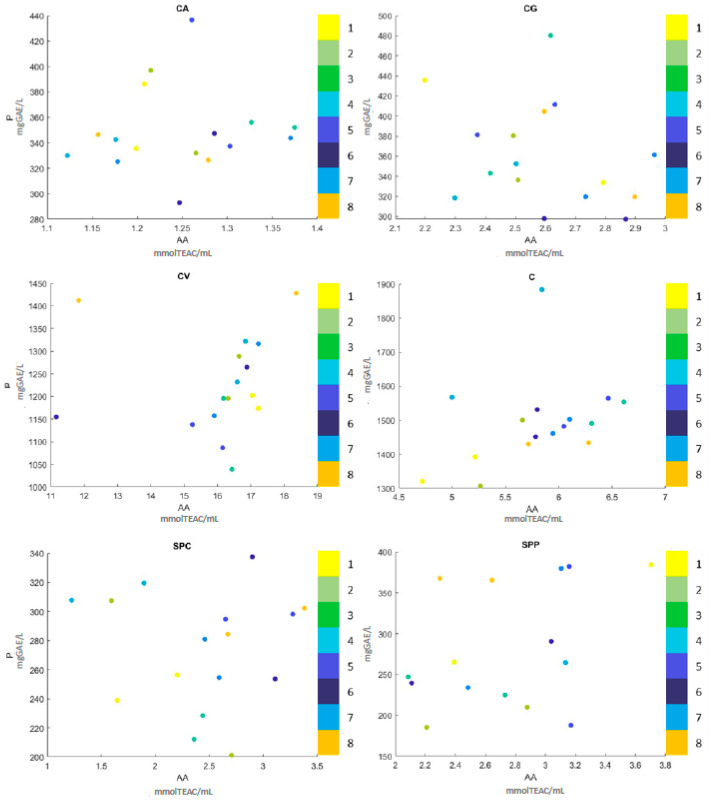
Results obtained for antioxidant activity (AA) and polyphenol content (P) divided by substrate. The spectrum shows the colors corresponding to each combination.

In order to study the nutritional features of the tomato-based products obtained with the selected combinations, antioxidant activity (DPPH) and total polyphenols content (Folin–Ciocâlteu) were determined (Table 2). Figure 6 reported a scatter plot of data referring to fermented samples, refrigerated and pasteurized. Even if the comparison with the control and between samples could be more easy detectable by Table 2, Figure 6 offers a clear picture of the best combination for each substrate.

Differences were observed among samples. The increase of the antioxidant activity was observed in CG extracts but particularly in tomato juices (SPC and SPP). Overall, polyphenols generally increased after fermentation in the same samples and in some CA-fermented products, especially when stabilized with refrigeration. This tendency in increasing polyphenols content and antioxidant activity in fermented tomato juices is in agreement with the data reported by Liu et al. [29].

The different performance of the combinations employed allowed to select the best parameters to be used in each product, which are easily identified in the scatter plot (Figure 6).

In general, in CA, differences in the polyphenol content were observed due to the stabilization treatment (NTT or TT) after fermentation in samples no. 1, 2, 5 and 6 termically treated where there was a decrease (Table 2). The best condition for CA-fermented products was the combination no. 5 stabilized with refrigeration. In this case, the final product was characterized by higher antioxidant activity and the highest content of polyphenols, which reached 436.57 ± 0.57 mgGAE/L. Studies on fermented beverage added with tomato juices show that the increase in antioxidant activity after fermentation is due to the high content of lycopene and other phenolic compounds present in the fruit [30,31].

Among the fermented CG products, sample no. 3 seems to be the best combination with higher polyphenol content and antioxidant activity.

Interestingly, the strain *L. rhamnosus*, used in combination no. 3 and no. 6, showed a different behavior, in term of polyphenol content (Table 2 and Figure 6). Despite the fact that microbial growth was not relevant for both samples, combination no. 3 showed a high level of polyphenols (480.29 ± 2.29). Similar results were obtained by Ricci et al. [20], who reported an increase in bioactive molecules even where the growth of *Lactobacillus* was absent.

The last tomato extract, CV, had a high antioxidant activity and polyphenols concentration even without fermentation. Except for combination no. 8, fermentation has not changed the antioxidant activity and polyphenols content (Table 2). This sample, together with the combination no. 3 was based on the use of the co-colture of *Pediococcus acidilactici* and *L. rhamnosus*, and differ for the inoculum level. For both samples, no growth of these LAB was noted. To note, combination no. 8 showed the highest concentration of polyphenols, in comparison to the control, reaching 1428.44 ± 1.92 mgGAE/L (Table 2 and Figure 6). A similar value was observed also in the pasteurized sample (Table 2).

Fermented tomato puree (C), where a considerable microbial increase was noted, showed a lower value of antioxidant activity and polyphenols content, in comparison to the control. To note, after the application of thermal treatment, we observed an increase of polyphenols and antioxidant activity (combination no. 2 and no. 4), as already seen in some CG and CG samples (Table 2). Although the heating process have negative effects on phenolic content, some authors suggested the opposite. Nicoli et al. [32] reported that heat treatment could lead to the formation of new compounds with increased antioxidant properties. Moreover, Van het Hof et al. [33] found that heat treatment can have a deleterious effect on the micronutrient content of vegetables but, at the same time, increase the bioavailability of some nutrients.

Analysis of the antioxidant activity revealed a slight increase, in comparison to the control, in almost all refrigerated and in some pasteurized samples (Table 1). The modulation of antioxidant activity of tomatoes juice after fermentation was already reported by Di Cagno et al. [24]. They observed that the employment of autochtonous strains better maintain or increase the antioxidant acivity compared to non autochtonous. On the contrary, in the present study, the employment of dairy strains, in specific conditions (inoculum size and fermentation time), can posistively modulate the antioxidant capacity and the total phenolic concentration. As reported in previous studies, fermentation can positively affect the nutritional characteristics, modulating the phenolic content and the content of acids or sugars [24,34,35]. In this study, we applied two different types of stabilization to open up different application perspectives: develop fermented tomato-based products with viable cells and potential bioactive metabolites (refrigerated samples) or develop fermented tomato-based products with potential bioactive metabolites (pasteurized samples).

Considering the results obtained, some substrates, such as orange extract (CA), yellow extract (CG) and tomato juice with “pizzutello” variety (SPC), demonstrated more suitabillity for bacterial growth thus for refrigerated drink preparation. In particular, the use of combination no. 5 for orange extract and combination no. 7 for pizzutello tomato juice allowed a product rich in viable cells and metabolites to be obtained. By contrast, in order to develop a pasteurized tomato drink, combination no. 8 in the pizzutello tomato juice sample can lead to pasteurized products with improved properties.

## 4. Conclusions

This study explored the use of a wide set of LAB strains of dairy origin and fermentation conditions aimed to develop a fermented tomato-based drink with good nutritional characteristics.

As expected, the nutritional features of tomato products, such as antioxidant activity and polyphenol content, firstly depend on the composition substrates. In this context, violet extract (CV) and tomato puree (C) appear to be the most interesting, especially for polyphenol concentration. Starting from these observations, we have monitored the contribution, in function of the combinations used, of fermentation and the effect of refrigeration or pasteurization. A significant microbial growth was recorded in all samples, except for violet extract, but it was not always related to the improvement of antioxidant activity and polyphenol content.

This is the case of tomato puree, which appears unuitable for lactic acid fermentation. For the other tomato-based substrates, at least one combination was able to increase nutritional profile. Despite the requirement for future studies on this topic, which should aim to carry out the management of an effective technology transfer and to evaluate the products during storage, the results of this study highlight the possibility of formulating new beverages fermented with LAB of dairy origin characterized by high antioxidant activity and polyphenolic content.

## Figures and Tables

**Table 1:** Composition of tomato extract juice.

CA	CG	CV
Orange tomato juice 28%, Williams pear puree 22.3%, white grape juice 16%, carrot juice 14.2%, orange juice 13.8%, ginger puree 0.8%	Yellow tomato juice 27%, Golden Delicious apple puree 24%, white grape juice 24%, Williams pear puree 10%, yellow carrot juice 10%, lemon juice 0.1%	Tomato juice 27%, strawberry puree 14.6%, pomegranate juice 14.6%, white grape juice 14.6%, Golden Delicious apple puree 14.6%, purple carrot juice 13.6%

**Table 2:** Results from antioxidant activity (AA) and quantification of total polyphenols (P). Average value ± standard deviation are reported for refrigerated (NTT) and pasteurized samples (TT) with regard to the control unfermented (NF). * significant (*p* < 0.05) differences in comparison with unfermented sample.

		AA (mmolTEAC/mL)		P (mgGAE/L)	
		NTT	TT	NTT	TT
**CA**	NF	1.23 ± 0.02		347.90 ± 1.47	
	1	1.21 ± 0.02	1.20 ± 0.02	386.38 ± 2.59 *	335.62 ± 0.72 *
	2	1.21 ± 0.02	1.27 ± 0.02	396.95 ± 0.33 *	332.00 ± 0.76 *
	3	1.37 ± 0.02 *	1.33 ± 0.02 *	352.19 ± 0.44 *	356.19 ± 0.44 *
	4	1.12 ± 0.02 *	1.18 ± 0.03	330.00 ± 0.76 *	342.67 ± 0.16 *
	5	1.26 ± 0.03	1.30 ± 0.01 *	436.57 ± 0.57 *	337.43 ± 0.49 *
	6	1.29 ± 0.02	1.25 ± 0.01	347.52 ± 1.94	292.95 ± 2.40 *
	7	1.18 ± 0.01 *	1.37 ± 0.02 *	325.33 ± 2.03 *	343.81 ± 1.15 *
	8	1.28 ± 0.01 *	1.16 ± 0.01 *	326.38 ± 0.66 *	346.57 ± 6.96
**CG**	NF	2.12 ± 0.02		324.57 ± 1.51	
	1	2.20 ± 0.03 *	2.79 ± 0.04 *	435.71 ± 2.00 *	333.71 ± 1.31 *
	2	2.49 ± 0.25 *	2.51 ± 0.02	380.48 ± 0.33 *	336.29 ± 0.49 *
	3	2.62 ± 002 *	2.42 ± 0.02 *	480.29 ± 2.29 *	343.14 ± 2.27 *
	4	2.50 ± 0.01 *	2.30 ± 0.06 *	352.48 ± 1.41 *	318.38 ± 0.59 *
	5	2.37 ± 0.02 *	2.63 ± 0.02 *	381.33 ± 0.66 *	411.43 ± 0.29 *
	6	2.87 ± 0.10 *	2.60 ± 0.06 *	297.24 ± 3.00 *	297.90 ± 3.06 *
	7	2.73 ± 0.08 *	2.96 ± 0.03 *	319.62 ± 0.59 *	361.33 ± 6.58 *
	8	2.60 ± 0.01 *	2.90 ± 0.11 *	404.67 ± 1.94 *	319.62 ± 2.50
**CV**	NF	17.10 ± 0.07		1366.32 ± 17.31	
	1	17.05 ± 0.19	17.23 ± 0.09	1202.22 ± 2.69 *	1173.52 ± 3.38 *
	2	16.32 ± 0.08 *	16.65 ± 0.10 *	1195.33 ± 0.67 *	1288.81 ± 1.72 *
	3	16.44 ± 0.11 *	16.18 ± 0.14 *	1038.89 ± 1.39 *	1195.43 ± 0.40 *
	4	16.60 ± 0.18 *	16.84 ± 0.14	1231.78 ± 0.77 *	1321.92 ± 0.68 *
	5	16.16 ± 0.13 *	15.26 ± 0.01 *	1086.44 ± 0.38 *	1137.67 ± 0.68 *
	6	16.89 ± 0.13	11.16 ± 0.09 *	1264.89 ± 0.38 *	1154.79 ± 0.68 *
	7	17.23 ± 0.17	15.91 ± 0.15 *	1316.44 ± 0.38 *	1157.31 ± 0.40 *
	8	18.37 ± 0.15 *	11.84 ± 0.17 *	1428.44 ± 1.92 *	1412.33 ± 0.01 *
**C**	NF	7.21 ± 0.08		2354.89 ± 4.56	
	1	5.21 ± 0.05 *	4.72 ± 0.16 *	1392.33 ± 1.10 *	1319.82 ± 4.11 *
	2	5.26 ± 0.14 *	5.66 ± 0.21 *	1306.48 ± 0.84 *	1500.09 ± 0.84 *
	3	6.61 ± 0.01 *	6.31 ± 0.12 *	1553.61 ± 3.57 *	1490.59 ± 0.32 *
	4	5.00 ± 0.15 *	5.84 ± 0.08 *	1567.31 ± 3.85 *	1884.20 ± 0.63 *
	5	6.46 ± 0.05 *	6.05 ± 0.14 *	1564.20 ± 3.65 *	1482.01 ± 5.62 *
	6	5.80 ± 0.09 *	5.78 ± 0.11 *	1531.32 ± 1.14 *	1451.51 ± 5.28 *
	7	6.10 ± 0.09 *	5.94 ± 0.08 *	1502.47 ± 4.68 *	1461.37 ± 0.55 *
	8	6.28 ± 0.04 *	5.72 ± 0.07 *	1433.42 ± 1.45 *	1430.14 ± 1.98 *
**SPC**	NF	1.59 ± 0.13		263.14 ± 0.43	
	1	2.20 ± 0.36	1.65 ± 0.08	256.48 ± 4.30	239.00 ± 0.14 *
	2	1.59 ± 0.05	2.71 ± 0.23 *	307.52 ± 0.82 *	201.19 ± 0.22 *
	3	2.44 ± 0.05 *	2.36 ± 0.25 *	228.48 ± 0.16 *	212.24 ± 0.22 *
	4	1.90 ± 0.18	1.23 ± 0.03 *	319.62 ± 0.87 *	307.90 ± 0.33 *
	5	2.65 ± 0.01 *	3.27 ± 0.49 *	294.76 ± 1.29 *	298.29 ± 0.89 *
	6	3.11 ± 0.08 *	2.90 ± 0.17 *	253.71 ± 2.00 *	337.48 ± 0.08 *
	7	2.46 ± 0.08 *	2.59 ± 0.16 *	281.05 ± 4.52 *	254.62 ± 0.50 *
	8	2.67 ± 0.17 *	3.38 ± 0.09 *	284.48 ± 0.16 *	302.33 ± 0.08 *
**SPP**	NF	2.38 ± 0.06		265.81 ± 0.59	
	1	3.70 ± 0.19 *	2.39 ± 0.19	384.19 ± 5.75 *	265.14 ± 0.29
	2	2.88 ± 0.20	2.21 ± 0.21	209.90 ± 0.16 *	185.43 ± 0.01 *
	3	2.73 ± 0.01 *	2.09 ± 0.05 *	224.95 ± 1.29 *	247.05 ± 0.16 *
	4	3.13 ± 0.12 *	3.13 ± 0.12 *	264.57 ± 0.01	264.57 ± 0.01
	5	3.16 ± 0.10 *	3.17 ± 0.07 *	382.19 ± 0.44 *	187.71 ± 0.76 *
	6	3.04 ± 0.19 *	2.11 ± 0.18	290.57 ± 0.29 *	239.62 ± 0.33 *
	7	3.11 ± 0.02 *	2.48 ± 0.10	379.62 ± 0.92 *	234.10 ± 0.33 *
	8	2.30 ± 0.11	2.64 ± 0.07 *	367.62 ± 0.17 *	365.43 ± 0.86 *

## References

[1] Di Cagno R., Coda R., De Angelis M., Gobbetti M. (2013). Exploitation of vegetables and fruits through lactic acid fermentation. Food Microbiol..

[2] Rimm E.B., Ascherio A., Giovannucci E., Spiegelman D., Stampfer M.J., Willett W.C., Rimm N., Giovannucci M. (1996). Vegetable, Fruit, and Cereal Fiber Intake and Risk of Coronary Heart Disease Among Men. JAMA.

[3] Joshipura K.J., Ascherio A., Manson J.A.E., Stampfer M.J., Rimm E.B., Speizer F.E., Hennekens C.H., Spiegelman D., Willett W.C. (1999). Fruit and vegetable intake in relation to risk of ischemic stroke. J. Am. Med. Assoc..

[4] Welman A.D., Ullrich M. (2009). Exploitation of exopolysaccharides from lactic acid bacteria. Bacterial Polysaccharides: Current Innovations and Future Trends.

[5] Holzapfel W.H., Wood B.B.J. (2014). Introduction to the LAB. Lactic Acid Bacteria: Biodiversity and Taxonomy.

[6] Lynch K.M., Zannini E., Coffey A., Arendt E.K. (2018). Lactic Acid Bacteria Exopolysaccharides in Foods and Beverages: Isolation, Properties, Characterization, and Health Benefits. Annu. Rev. Food Sci. Technol..

[7] Di Cagno R., De Angelis M., Calasso M., Gobbetti M. (2011). Proteomics of the bacterial cross-talk by quorum sensing. J. Proteom..

[8] Espirito-Santo A.P., Carlin F., Renard C.M.G.C. (2015). Apple, grape or orange juice: Which one offers the best substrate for lactobacilli growth?—A screening study on bacteria viability, superoxide dismutase activity, folates production and hedonic characteristics. Food Res. Int..

[9] Papadimitriou K., Alegría Á., Bron P.A., De Angelis M., Gobbetti M., Kleerebezem M., Lemos J.A., Linares D.M., Ross P., Stanton C. (2016). Stress Physiology of Lactic Acid Bacteria. Microbiol. Mol. Biol. Rev..

[10] Filannino P., Cardinali G., Rizzello C.G., Buchin S., De Angelis M., Gobbetti M., Di Cagno R. (2014). Metabolic responses of Lactobacillus plantarum strains during fermentation and storage of vegetable and fruit juices. Appl. Environ. Microbiol..

[11] Ricci A., Cirlini M., Maoloni A., Del Rio D., Calani L., Bernini V., Galaverna G., Neviani E., Lazzi C. (2019). Use of dairy and plant-derived lactobacilli as starters for cherry juice fermentation. Nutrients.

[12] Filannino P., Bai Y., Di Cagno R., Gobbetti M., Gänzle M.G. (2015). Metabolism of phenolic compounds by Lactobacillus spp. during fermentation of cherry juice and broccoli puree. Food Microbiol..

[13] Dorais M., Ehret D.L., Papadopoulos A.P. (2008). Tomato (Solanum lycopersicum) health components: From the seed to the consumer. Phytochem. Rev..

[14] Stahl W., Sies H. (2005). Bioactivity and protective effects of natural carotenoids. Biochim. Biophys. Acta.

[15] Rissanen T.H., Voutilainen S., Nyyssönen K., Lakka T.A., Sivenius J., Salonen R., Kaplan G.A., Salonen J.T. (2001). Low serum lycopene concentration is associated with an excess incidence of acute coronary events and stroke: The Kuopio Ischaemic Heart Disease Risk Factor Study. Br. J. Nutr..

[16] Sesso H.D., Liu S., Gaziano J.M., Buring J.E. (2003). Dietary Lycopene, Tomato-Based Food Products and Cardiovascular Disease in Women. J. Nutr..

[17] Ricci A., Bernini V., Maoloni A., Cirlini M., Galaverna G., Neviani E., Lazzi C. (2019). Vegetable by-product lacto-fermentation as a new source of antimicrobial compounds. Microorganisms.

[18] Brand-Williams W., Cuvelier M.E., Berset C. (1995). Use of a free radical method to evaluate antioxidant activity. LWT Food Sci. Technol..

[19] Singleton V.L., Orthofer R., Lamuela-Raventòs R.M. (1999). Analysis of total phenols and other oxidation substrates and antioxidants by means of folin-ciocalteu reagent. Methods Enzym..

[20] Ricci A., Levante A., Cirlini M., Calani L., Bernini V., Del Rio D., Galaverna G., Neviani E., Lazzi C. (2018). The influence of viable cells and cell-free extracts of lactobacillus casei on volatile compounds and polyphenolic profile of elderberry juice. Front. Microbiol..

[21] Cirlini M., Ricci A., Galaverna G., Lazzi C. (2020). Application of lactic acid fermentation to elderberry juice: Changes in acidic and glucidic fractions. LWT Food Sci. Technol..

[22] Ricci A., Cirlini M., Calani L., Bernini V., Neviani E., Del Rio D., Galaverna G., Lazzi C. (2019). In vitro metabolism of elderberry juice polyphenols by lactic acid bacteria. Food Chem..

[23] Ricci A., Cirlini M., Levante A., Dall’Asta C., Galaverna G., Lazzi C. (2018). Volatile profile of elderberry juice: Effect of lactic acid fermentation using L. plantarum, L. rhamnosus and L. casei strains. Food Res. Int..

[24] Di Cagno R., Surico R.F., Paradiso A., De Angelis M., Salmon J.C., Buchin S., De Gara L., Gobbetti M. (2009). Effect of autochthonous lactic acid bacteria starters on health-promoting and sensory properties of tomato juices. Int. J. Food Microbiol..

[25] Koh J.H., Kim Y., Oh J.H. (2010). Chemical characterization of tomato juice fermented with bifidobacteria. J. Food Sci..

[26] Juven B.J., Weisslowicz H. (1981). Chemical Changes in Tomato Juices Caused by Lactic Acid Bacteria. J. Food Sci..

[27] Kaur S., Kaur H.P., Grover J. (2016). Fermentation of Tomato juice by Probiotic Lactic acid bacteria. Int. J. Adv. Pharm. Biol. Chem..

[28] Viskelis P., Juodeikiene G., Urbonaviciene D., Bartkiene E., Vidmantiene D. (2013). Lactic Acid Fermentation of Tomato: Effects on cis/trans Lycopene Isomer Ratio, β-Carotene Mass Fraction and Formation of L(+)- and D(–)-Lactic Acid. Food Technol. Biotechnol..

[29] Liu Y., Chen H., Chen W., Zhong Q., Zhang G., Chen W. (2018). Beneficial Effects of Tomato Juice Fermented by Lactobacillus Plantarum and Lactobacillus Casei: Antioxidation, Antimicrobial Effect, and Volatile Profiles. Molecules.

[30] Nursiwi A., Nurhartadi E., Utami R., Sari A.M., Laksono P.W., Aprilia E.N. (2017). Characteristic of Fermented Whey Beverage with Addition of Tomato Juice (Lycopersicum esculentum). IOP Conf. Ser. Mater. Sci. Eng..

[31] Wang K., Ma C., Gong G., Chang C. (2019). Fermentation parameters, antioxidant capacities, and volatile flavor compounds of tomato juice-skim milk mixtures fermented by Lactobacillus plantarum ST-III. Food Sci. Biotechnol..

[32] Nicoli M.C., Anese M., Manzocco L. (1999). Oil stability and antioxidant properties of an oil tomato food system as affected by processing. Adv. Food Sci..

[33] Van Het Hof K.H., de Boer B.C.J., Tijburg L.B., Lucius B.R., Zijp I., West C.E., Hautvast J.G.A.J., Weststrate J.A. (2000). Carotenoid Bioavailability in Humans from Tomatoes Processed in Different Ways Determined from the Carotenoid Response in the Triglyceride-Rich Lipoprotein Fraction of Plasma after a Single Consumption and in Plasma after Four Days of Consumption. J. Nutr..

[34] Hervert-Hernández D., Pintado C., Rotger R., Goñi I. (2009). Stimulatory role of grape pomace polyphenols on Lactobacillus acidophilus growth. Int. J. Food Microbiol..

[35] García-Ruiz A., Bartolomé B., Martínez-Rodríguez A.J., Pueyo E., Martín-Alvarez P.J., Moreno-Arribas M. (2008). V Potential of phenolic compounds for controlling lactic acid bacteria growth in wine. Food Control.

